# Mediolateral footpath stabilization during walking in people following stroke

**DOI:** 10.1371/journal.pone.0208120

**Published:** 2018-11-29

**Authors:** Pei-Chun Kao, Shraddha Srivastava

**Affiliations:** 1 Department of Physical Therapy, University of Massachusetts Lowell, Lowell, Massachusetts, United States of America; 2 Department of Health Sciences and Research, Medical University of South Carolina, Charleston, South Carolina, United States of America; Nanyang Technological University, SINGAPORE

## Abstract

Community dwelling stroke survivors most often fall while walking. Understanding how post-stroke individuals control mediolateral footpath during walking may help elucidate the mechanisms that contribute to walking instability. By applying the Uncontrolled Manifold (UCM) approach, we investigated (1) how post-stroke individuals coordinate lower-extremity joint motions to stabilize mediolateral footpath of the swing leg, and (2) how the inter-joint coordination in footpath stabilization correlates to their walking stability. Nine stroke subjects and nine healthy controls walked on a treadmill at four different speeds. UCM analysis partitions the variance of kinematic configurations across gait cycles into “good variance” (i.e., the variance component leading to a consistent footpath) or “bad variance” (i.e., the variance component leading to an inconsistent footpath). We found that both groups had a significantly greater “good” than “bad” variance (p<0.05) for most of the swing phase, suggesting that mediolateral footpath is an important variable stabilized by the central nervous system during walking. Stroke subjects had significantly greater relative variance difference (ΔV) (i.e. normalized difference between “good” and “bad” variance) (p<0.05), indicating a stronger kinematic synergy in footpath stabilization, than the controls. In addition, the kinematic synergy in mediolateral footpath stabilization is strongest during mid-swing but weakest during late swing in healthy gait. However, this phase-dependent strategy is preserved for mid-swing but not for late swing in stroke gait. Moreover, stroke and healthy subjects demonstrated different relationships between UCM and walking stability measures. A stronger kinematic synergy in healthy gait is associated with better walking stability whereas having more “good variance” or stronger kinematic synergy in stroke gait is associated with less walking stability. The current findings suggest that walking with too much “good variance” in people following stroke, despite no effect on the footpath, may adversely affect their walking stability to some extent.

## Introduction

Falls and fall-related injuries cause extremely costly health problems in stroke population [1]. Community-dwelling post-stroke individuals most often fall while walking [1, 2]. It was shown that post-stroke individuals were more unstable especially in the frontal plane during walking such as having greater dynamic instability, increased trunk sways, and asymmetric foot placement in the mediolateral direction [3–5]. In addition, post-stroke individuals also had greater variability in spatiotemporal gait measures than neurologically intact controls [4, 6]. Stroke-related impairments such as reduced sensorimotor function, insufficient muscle strength and elevated reflex responses [7] may all contribute to the unstable walking patterns. However, the underlying mechanisms of increased walking instability and gait variability following stroke are not fully understood.

Human walking is dynamically unstable in the mediolateral direction and requires active feedback control produced by the central nervous system (CNS) to maintain lateral balance [8–10]. A computational walking model [11] and the findings from the human experiments [8–10] suggested that adjusting mediolateral foot placement is an effective strategy to maintain walking stability in the frontal plane (i.e., mediolateral direction). In addition, Rankin et al (2014) showed that greater swing-phase activity of the gluteus medius (i.e., hip abductor) was correlated with more lateral foot placement of the swing leg and increased trunk sways relative to the stance leg during both normal and perturbed walking in healthy adults [12]. However, the relationship between the hip abductor activity, trunk sways and mediolateral foot placement was shown to be somewhat disrupted in post-stroke individuals [13]. These results suggest that the CNS actively controls for mediolateral foot placement during walking but this capability is compromised following stroke. Being able to control the stride-to-stride, mediolateral foot placement during walking would require the CNS to control the mediolateral footpath for at least some part of the swing phase. Krishnan et al (2013) found that the mediolateral footpath was stabilized by a kinematic synergy throughout most of the swing phase in neurologically intact individuals [14]. Nevertheless, it is unknown if post-stroke individuals also use similar strategy to control their mediolateral footpath for maintaining lateral balance. Understanding how post-stroke individuals control their mediolateral footpath during walking compared to their healthy controls may help elucidate the control strategies used by the post-stroke individuals that contribute to unstable walking.

The uncontrolled manifold (UCM) approach has been used to understand how the CNS organizes or coordinates abundant degrees of freedom available to the nervous system (i.e., elemental variables) such as multiple configurations of joint motion or muscle activation to perform a motor task [15, 16]. The UCM hypothesis assumes that the CNS co-varies multiple elemental variables in a way so that the desired values of the task variable can be stabilized or maintained relatively consistently. According to the UCM hypothesis, when performing a motor task, the variance of elemental variables can be split into two components. The variance component that does not lead to an increased variability in task performance or changes in the values of the task variable (i.e., “good variance” or V_UCM_), reflects the flexibility of the CNS for motor task performance. The other component leads to an increased variability of the task variable (i.e., “bad variance” or V_ORT_).

Precise control of important task variables during different phases of the gait cycle has been demonstrated in the past [14, 17]. Previous literature suggests that foot trajectory in the vertical and anterior-posterior direction as well as in the mediolateral direction during walking is controlled by the CNS in neurologically intact individuals [14, 18, 19]. Following a neurological injury such as stroke, the CNS uses compensatory strategies to stabilize the performance of task variables during walking, resulting in altered motor coordination [19–21]. Previous studies demonstrated that although there is impaired coordination at the level of elemental variables (e.g., joint angle, muscle activation pattern and timing) following stroke, the CNS is still able to stabilize important task variables during walking [19, 20]. However, there is limited understanding of how the CNS adapts to the altered motor coordination following stroke to control mediolateral footpath, and how the control strategies used by the post-stroke individuals affects their walking stability.

The purpose of this study was to apply UCM approach to investigate the role of inter-joint coordination (i.e., kinematic synergy) in the mediolateral footpath stabilization of the swing leg during walking. Specifically, we examined how post-stroke individuals coordinate lower-extremity joint motions to stabilize the mediolateral footpath of their swing leg compared to neurologically intact individuals. Additionally, to enhance our understanding on the role of footpath control in walking stability, we investigated how the inter-joint coordination of footpath stabilization relates to their walking stability. In the current study, we analyzed inter-joint coordination of footpath stabilization in the same cohort of subjects tested previously for dynamic stability reported by Kao et al (2014) [4] and compared their kinematic synergy of footpath control with their walking stability. Based on the previous literature [19, 20, 22], we hypothesized that post-stroke individuals would still possess the capability of coordinating joint motions to stabilize mediolateral footpath during walking by showing a significantly greater amount of “good variance” compared to the bad variance. We expected that the kinematic synergy to stabilize the mediolateral footpath would be weaker in the post-stroke individuals compared to their healthy controls.

## Materials and method

### Participants

Nine chronic (> 6 months of post-stroke duration), post-stroke individuals (four female, five male, age: 60.8±9.0 years, post-stroke duration: 3.4±3.3 years, lower-extremity Fugl-Meyer score: 27±4) and their gender- and age-matched (±5 years) healthy controls (age: 61.7±10.0 years) gave written informed consent to participate in the study. This study complied with the Declaration of Helsinki and was approved by the Institutional Review Board of the University of Delaware (#275894). The exclusion criteria for the post-stroke subjects include more than one stroke, unable to walk independently for one minute continuously, moderate/severe chronic white matter disease or cerebellar stroke on MRI, neglect/hemianopia, history of lower extremity joint replacement, or any medical condition, other than stroke, that affects walking ability.

### Experimental protocol

All subjects walked on a treadmill at four different speeds: 60%, 80% and 100% of their preferred walking speed (PWS) and the fastest attainable speed (FAS) [4]. Each speed was tested three times in a pseudo-randomized order for 1 min or at least continuous 30 strides. Before the testing, a 5-min familiarization session of treadmill walking was administered [23].

### Data acquisition and analysis

Previously, we recorded 3-dimensional (3D) kinematic data using an eight-camera video system (120 Hz, Motion Analysis Corporation, Santa Rosa, CA, USA) with 46 reflective markers attached on the lower body, trunk and over the C7 vertebra. At each walking speed, we collected 3 trials and used 30 strides of data from each trial for data analysis [4]. We used commercial software (Visual3D, C-Motion Inc., Germantown, MD, USA) to derive ankle, knee and hip centers using marker positions. Data were then extracted for the swing phase for each of the legs (left swing-right stance and right swing-left stance) and time normalized to 100% of the swing phase. The mediolateral footpath of the swing leg (Foot_ML_) was expressed as the mediolateral position of the swing leg’s ankle joint center relative to the stance leg’s ankle joint center [14].

### UCM analysis

**Fig 1:**
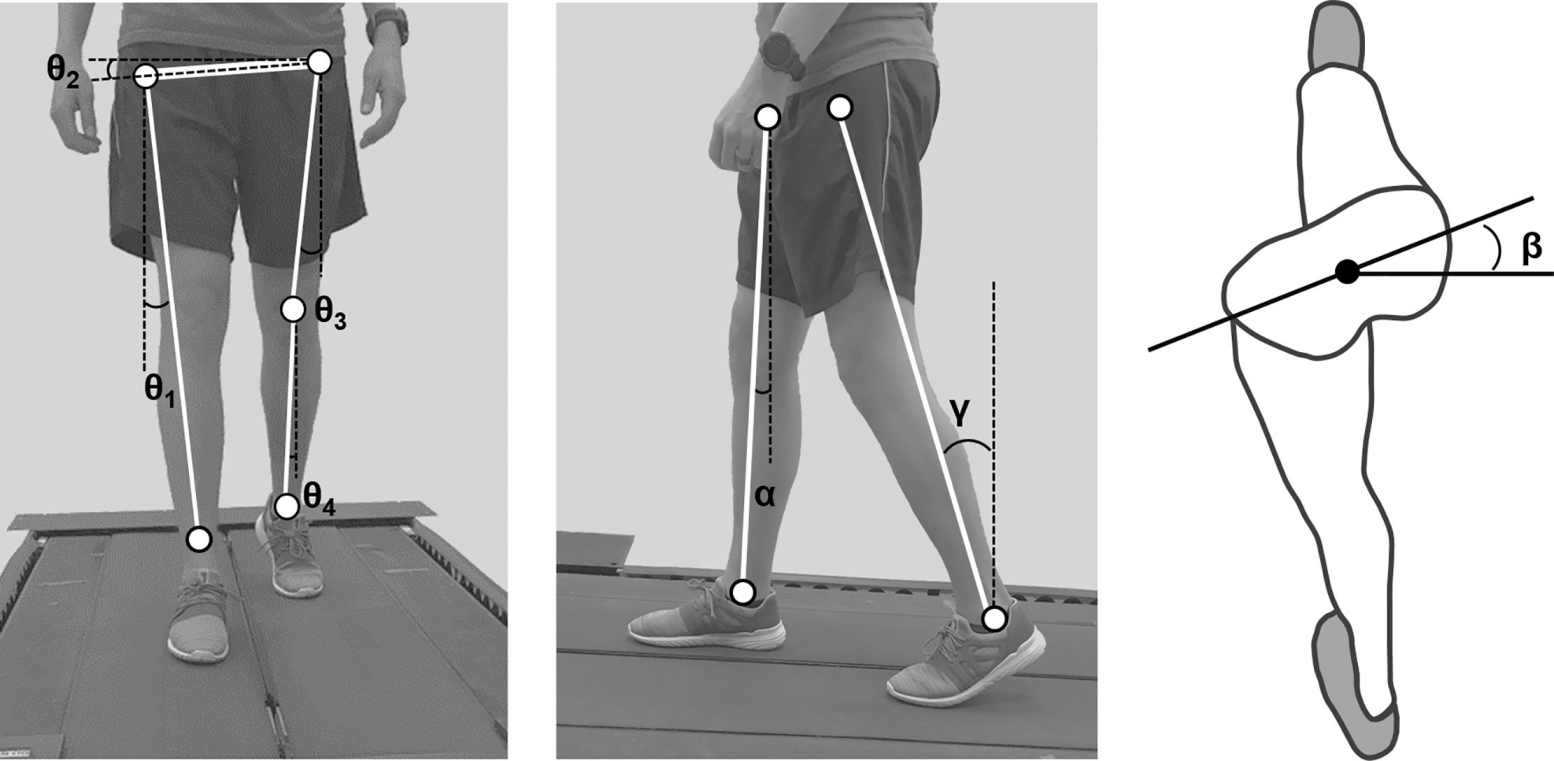
Segments (S_1-4_) and segment angles (θ_1–4_, α, β, γ) included in the geometric model. (a), (b) and (c) are the views from the frontal plane, sagittal plane and transverse plane, respectively.

Details of the UCM analysis can be found elsewhere [15, 20, 24]. A geometric model based on Krishnan et al (2013) [14] to derive the mediolateral footpath of the swing leg was first created. Briefly, the geometric model includes four segments: a stance leg (S_1_), pelvis (S_2_), swing-leg thigh (S_3_) and swing-leg shank (S_4_), with corresponding segment length of L_1-4_ (**Fig 1**). θ_1_, θ_3_ and θ_4_ are the angles between each of the segments (S_1_, S_3_, S_4_) and the vertical in frontal plane. θ_2_ is the angle between S_2_ and the horizontal in frontal plane. Since there is noticeable motion outside of the frontal plane during walking, we also included angles outside of the frontal plane (i.e. α, β, and γ) in the geometric model to account for the changes in the effective length of the segments that were projected onto the frontal plane. α is the angle between S_1_ and the vertical in sagittal plane, β is the angle between S_2_ and the horizontal in transverse plane, and γ is the angle between the swing leg and vertical in the sagittal plane.

$${{Foot}_{ML}=L}_{1}\;\cos\;\alpha \;\sin\theta_{1}+L_{2}\;\cos\;\beta \;\cos\;\theta_{2}+L_{3}\;\cos\;\gamma \;\sin\;\theta_{3}+L_{4}\;\sin\;\theta_{4}\Theta =[\theta_{1}\;\theta_{2}\;\theta_{3}\;\theta_{4}\;\alpha \;\beta \;\gamma ]$$ (1)

UCM analysis was performed at each normalized time point of the swing phase, across all steps, to determine how much of the variance of the kinematic segment configurations led to the footpath variability (V_ORT_) or reflected segment configurations that stabilized the footpath (V_UCM_). The Jacobian matrix (J), the matrix of partial derivatives of the task variable (i.e., Foot_ML_) with respect to the segment angles (θ_1–4_, α, β, γ), relates the changes in segment configurations to the changes in foot positions. J was defined as:

$$J=\left[\frac{\partial {Foot}_{ML}}{\partial \Theta }\right]=[L_{1}\;\cos\;\alpha \;\cos\;\theta_{1},-L_{2}\;\cos\;\beta \;\sin\;\theta_{2},L_{3}\;\cos\;\gamma \;\cos\theta_{3},L_{4}\;\cos\;\theta_{4},-L_{1}\;\sin\;\alpha \;\sin\;\theta_{1},-L_{2}\;\sin\;\beta \;\cos\;\theta_{2},-L_{3}\;\sin\;\gamma \;\sin\;\theta_{3}]$$ (2)

The linear approximation of UCM is based on the Jacobian at the reference configuration (mean segment angles across steps) i.e. $J(\bar{\Theta })$. The null space of this Jacobian is a set of solutions such that $J(\bar{\Theta })∙\varepsilon =0$. The basis vector (ε) i.e. the null space was computed at each normalized time point of the swing phase using MATLAB. Within the UCM subspace, all combinations of the segment configurations have no effect on the mediolateral foot positions. The space orthogonal to the UCM subspace (orthogonal space, ORT) represents the subspace where combinations of segment configurations result in changes in the mediolateral foot positions.

At each normalized time point of the swing phase, the deviation of each step’s segment configuration vector from the mean segment configuration was projected on to the null space of the Jacobian that is spanned by a set of (n-d) basis vectors:

$$\Theta_{UCM}=\sum_{i=1}^{n-d}(\varepsilon_{i}^{T}∙(\Theta -\bar{\Theta }))\varepsilon_{i}$$ (3)

and the space orthogonal to the null space (ORT):

$$\Theta_{ORT}=(\Theta -\bar{\Theta })-\Theta_{UCM}$$ (4)

In this study, n = 7 represents the number of dimensions of the segmental variables and d = 1 represents the number of dimensions of the task variable. The variances of these projections were then calculated. The variance in the segment configuration that did not affect the Foot_ML_ (V_UCM_) was computed as the average of the squared length of *Θ*_*UCM*_ across steps (N) and normalized by the degrees of freedom (DOFs) within the UCM subspace (n—d):

$$V_{UCM}=\frac{1}{\left(N\;steps\right)}\frac{1}{n-d}\sum_{i=1}^{N}\Theta_{UCM}^{2}$$ (5)

The variance in the segment configuration that affects the Foot_ML_ (V_ORT_) was computed as the average of the squared length of *Θ*_*ORT*_ across steps (N) and normalized by the DOFs within the orthogonal subspace (d):

$$V_{ORT}=\frac{1}{\left(N\;steps\right)}\frac{1}{d}\sum_{i=1}^{N}\Theta_{ORT}^{2}$$ (6)

The relative variance difference between V_UCM_ and V_ORT_ (ΔV) was computed as:

$$\Delta V=\frac{V_{UCM}-V_{ORT}}{V_{UCM}+V_{ORT}}$$ (7)

The relative variance difference (ΔV) reflects the strength of the kinematic synergy to stabilize the task variable. A value of ΔV closer to positive one indicates a stronger kinematic synergy, meaning many equivalent segment configurations, in using motor abundance to stabilize Foot_ML_ during walking. The total variance (V_TOT_) was computed as:

$$V_{TOT}=\frac{{(n-d)V}_{UCM}+dV_{ORT}}{n+d}$$ (8)

We divided the swing phase into three sub-phases: early swing (0–33%), mid-swing (34–67%), and late swing (68–100%). We averaged V_UCM_, V_ORT_, ΔV, and V_TOT_ across entire swing phase and across each of the sub-phases of swing, respectively, for each subject.

### Statistics

Mixed-design ANOVAs were then performed with within-subject factors (speed, variance components: V_UCM_ versus V_ORT_) and a between-subject factor (group) for the average V_UCM_ and V_ORT_ across entire swing and each of the sub-phases. We used separate mixed-design ANOVAs to test for differences in the average ΔV and V_TOT_ across entire swing and at each of the sub-phases with a within-subject factor (speed) and between-subject factor (group). To test for differences in average ΔV between the sub-phases of swing, we performed another mixed-design ANOVA with a within-subject factor (phase) and a between-subject factor (group). We set the significance level at *p*<0.05 and used Tukey Honestly Significant Difference (THSD) post hoc tests for pair-wise comparisons if a significant main effect or interaction effect of primary interests (e.g., group*variance component, group*phase) was detected. The effect size for each ANOVA component (i.e., main and interaction effect) and significant post-hoc comparison was estimated using partial eta squared (*η*^*2*^) and Cohen’s d, respectively [25–27]. Following Cohen and previous studies [25, 27, 28], *η*^*2*^ values were interpreted as: 0.02 “small” effect, 0.13 “medium” effect, and 0.26 “large” effect whereas Cohen’s d values were interpreted as: 0.2 “small” effect, 0.5 “medium” effect, and 0.8 “large” effect. Pearson’s correlations were used to assess the relationship between UCM measures (average V_UCM_, V_ORT_, ΔV, and V_TOT_ across entire swing) and the walking stability and variability data [4]. Following Cohen [29], Pearson’s correlation coefficient (r) values were interpreted as: 0.1 “small” effect, 0.3 “medium” effect, and 0.5 “large” effect. All statistical analyses were performed in JMP version 13.0.0 (SAS institute Inc., Cary, NC, USA).

The walking stability and variability measures for the correlation analyses included short-term local divergence exponent (LDE) and maximum Floquet multipliers (maxFM) for the mediolateral trunk motion [30], average and variability of mediolateral dynamic margins of stability (MOS_ML_) [31] and step width, and the mean standard deviations (meanSD) of the C7 marker positions and velocities across gait cycle in the mediolateral direction. A larger value of short-term LDE or maxFM indicates greater instability of mediolateral trunk motion represented by the C7 vertebral marker velocity profile. MOS_ML_ was computed as the lateral distances between the “velocity-adjusted” center of mass positions and the lateral toe marker of the leading foot at heel strikes. The meanSD of C7 marker mediolateral positions and velocities quantify overall variability of subject’s lateral displacements (i.e., drift) on the treadmill and stride-to-stride trunk movement variability, respectively.

## Results

### Overall swing phase

**Fig 2:**
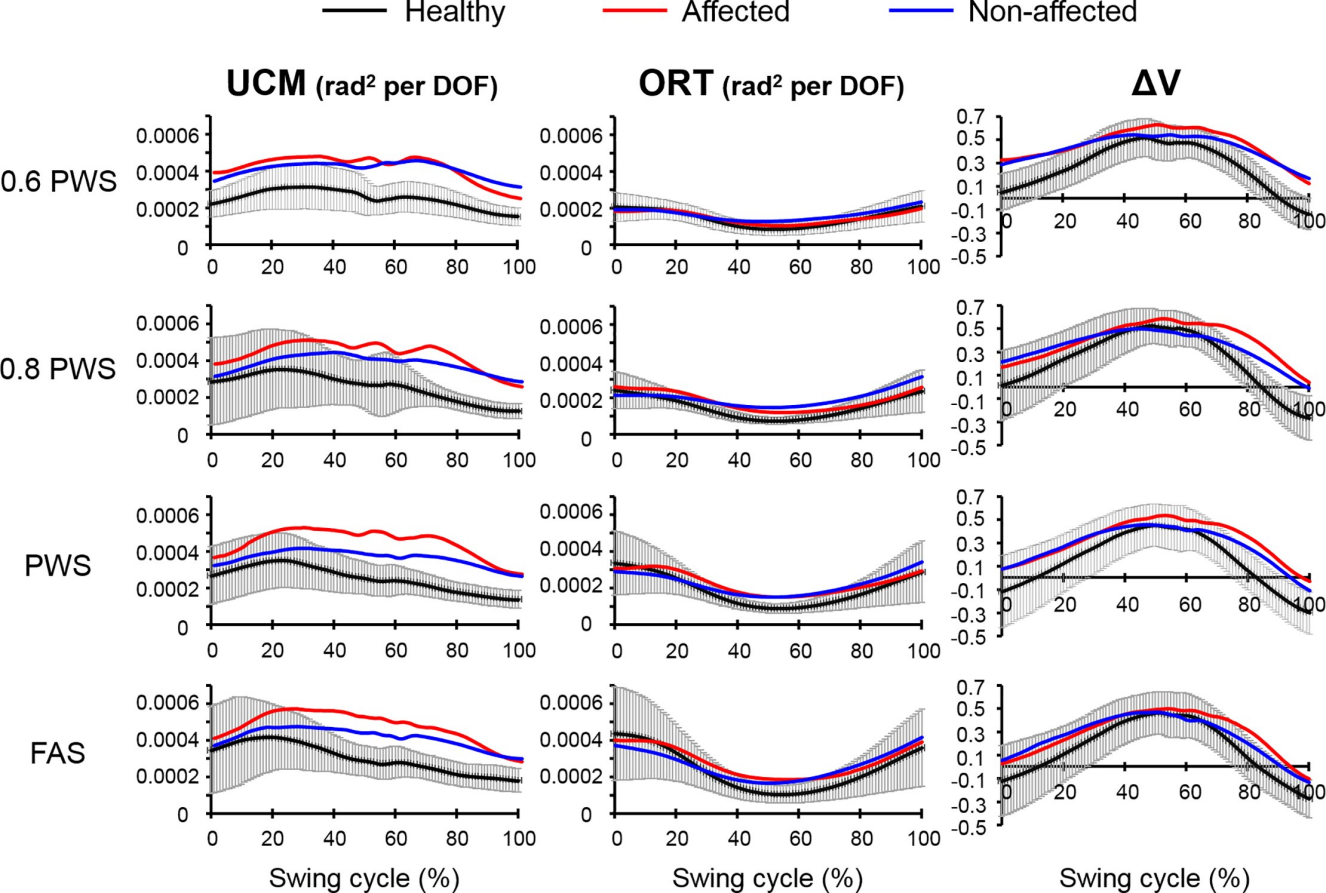
UCM variables across the entire swing phase for healthy controls (black line), stroke affected leg (red line), and stroke unaffected leg (blue line) at four different speeds which are 60%, 80% and 100% of their preferred walking speed (PWS) and the fastest attainable speed (FAS). Error bars (in grey) are ± 1 STD of data in healthy group.

**Fig 3:**
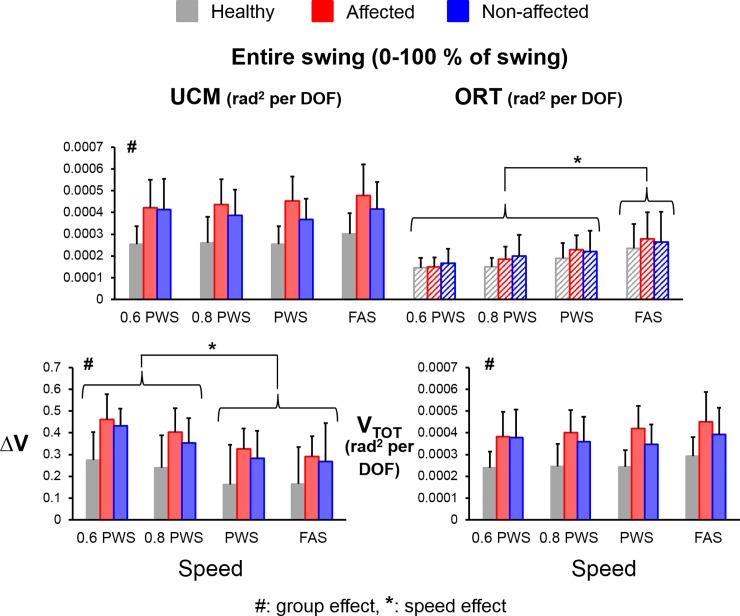
Average values of UCM variables across the entire swing phase (V_UCM-whole_, V_ORT-whole_, ΔV_whole_ and V_TOT-whole_) for healthy controls (grey bars), stroke affected leg (red bars), and stroke unaffected leg (blue bars). Error bars represent 1 STD. # indicates significant difference between stroke and control groups. * indicates significant difference between different speeds.

Overall, subjects had ΔV greater than zero, indicating V_UCM_ > V_ORT_, throughout majority of the swing phase (**Fig 2**). There were significant main effects for group (F_(1,112)_ = 15.74, *η*^*2*^ = 0.12, *p*<0.001, power = 0.98), variance component (V_UCM_ versus V_ORT_) (F_(1,112)_ = 68.65, *η*^*2*^ = 0.38, *p*<0.001, power = 1.00), and speed (F_(3,112)_ = 7.83, *η*^*2*^ = 0.17, *p*<0.001, power = 0.99) as well as a significant interaction effect for group*variance component (F_(1,112)_ = 11.76, *η*^*2*^ = 0.10, *p*<0.001, power = 0.93). Both groups had significantly greater average V_UCM_ compared to average V_ORT_ across the entire swing (V_UCM-whole_ > V_ORT-whole_) (THSD post hoc, *p*<0.05, stroke UCM-ORT: Cohen’s d = 3.90, healthy UCM-ORT: Cohen’s d = 1.62) (**Fig 3**), suggesting that mediolateral footpath was stabilized during the swing phase of walking. For the group effect, stroke subjects had significantly greater V_UCM-whole_ compared to healthy controls (THSD post hoc, *p*<0.05, UCM stroke-healthy: Cohen’s d = 2.47). However, there was no group effect for V_ORT-whole_, indicating that stroke subjects did not have greater amount of variance in the segment configuration that affects the mediolateral foot positions during swing (Foot_ML_).

Stroke subjects also demonstrated significantly greater average relative variance difference across the entire swing phase (ΔV_whole_) compared to healthy controls (main group effect, F_(1,48)_ = 42.91, *η*^*2*^ = 0.47, *p*<0.001, power = 1.00). These results indicate that stroke subjects used stronger kinematic synergy to stabilize Foot_ML_ during walking than the healthy subjects. Stroke subjects also had significantly greater average total variance across entire swing (V_TOT-whole_) than the healthy controls (main group effect, F_(1,48)_ = 29.39, *η*^*2*^ = 0.38, *p*<0.001, power = 1.00). For the speed effect, subjects had significantly greater V_ORT-whole_ at the fastest attainable speed (FAS) than at 60%, 80% and 100% of their preferred walking speeds (PWS) (THSD post hoc, *p*<0.05, Cohen’s d = 2.05, 1.66 and 0.94, respectively). There was no main speed effect for the V_UCM-whole_ or V_TOT-whole_. Accordingly, subjects had significantly greater ΔV_whole_ at the lower speeds (60% PWS and 80% PWS) than at the higher speeds (PWS and FAS) (THSD post hoc, *p*<0.05, all Cohen’s d > 1.34).

### Early swing

**Fig 4:**
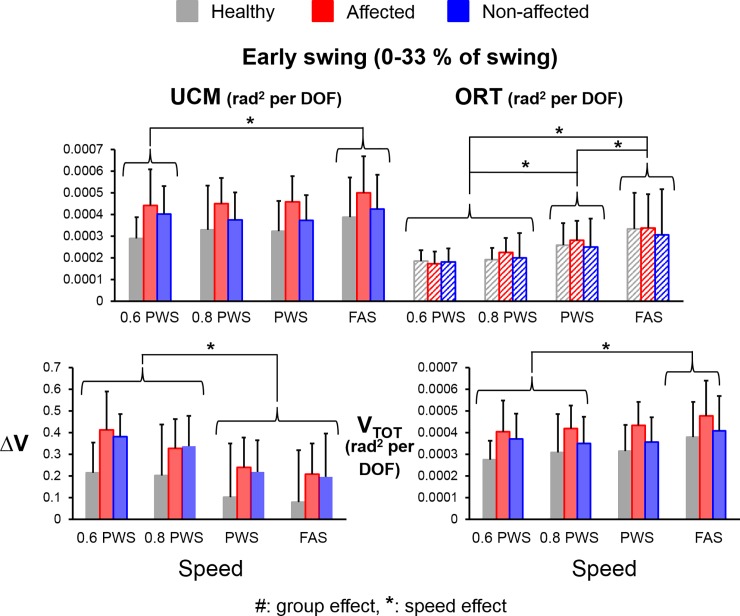
Average values of UCM variables during the early swing (V_UCM-early_, V_ORT-early_, ΔV_early_ and V_TOT-early_) for healthy controls (grey bars), stroke affected leg (red bars), and stroke unaffected leg (blue bars). Error bars represent 1 STD. * indicates significant difference between different speeds.

There were significant main effects for variance component (V_UCM_ versus V_ORT_) (F_(1,112)_ = 24.64, *η*^*2*^ = 0.18, *p*<0.001, power = 0.99) and speed (F_(3,112)_ = 8.57, *η*^*2*^ = 0.19, *p*<0.001, power = 0.99) as well as a significant interaction effect for group*variance component (F_(1,112)_ = 4.61, *η*^*2*^ = 0.04, *p* = 0.03, power = 0.57). Both groups had significantly greater average V_UCM_ compared to average V_ORT_ during the early swing (V_UCM-early_ > V_ORT-early_) (THSD post hoc, *p*<0.05, stroke UCM-ORT: Cohen’s d = 2.37, healthy UCM-ORT: Cohen’s d = 0.94) (**Fig 4**). There was no group effect for average V_UCM_, V_ORT_, ΔV or V_TOT_ during the early swing (all *p*>0.05), suggesting that stroke subjects walked with similar control strategy as the healthy controls during the early swing. For the speed effect, subjects had significantly greater V_UCM-early_ at the FAS than at 60% of PWS (THSD post hoc, *p*<0.05, Cohen’s d = 0.89). In addition, subjects also had significantly greater V_ORT-early_ at the higher speeds than at the lower speeds (FAS > PWS > 80% PWS = 60% PWS) (THSD post hoc, *p*<0.05, Cohen’s d > 0.84 for all significant pairs). Correspondingly, subjects demonstrated significantly greater ΔV_early_ at the lower speeds (60% PWS and 80% PWS) than at the higher speeds (PWS and FAS) (THSD post hoc, *p*<0.05, Cohen’s d >1.37 for all significant pairs) but significantly greater V_TOT-early_ at the FAS than at the 60% and 80% of PWS (THSD post hoc, *p*<0.05, Cohen’s d = 1.10 and 0.84, respectively).

### Mid-swing

**Fig 5:**
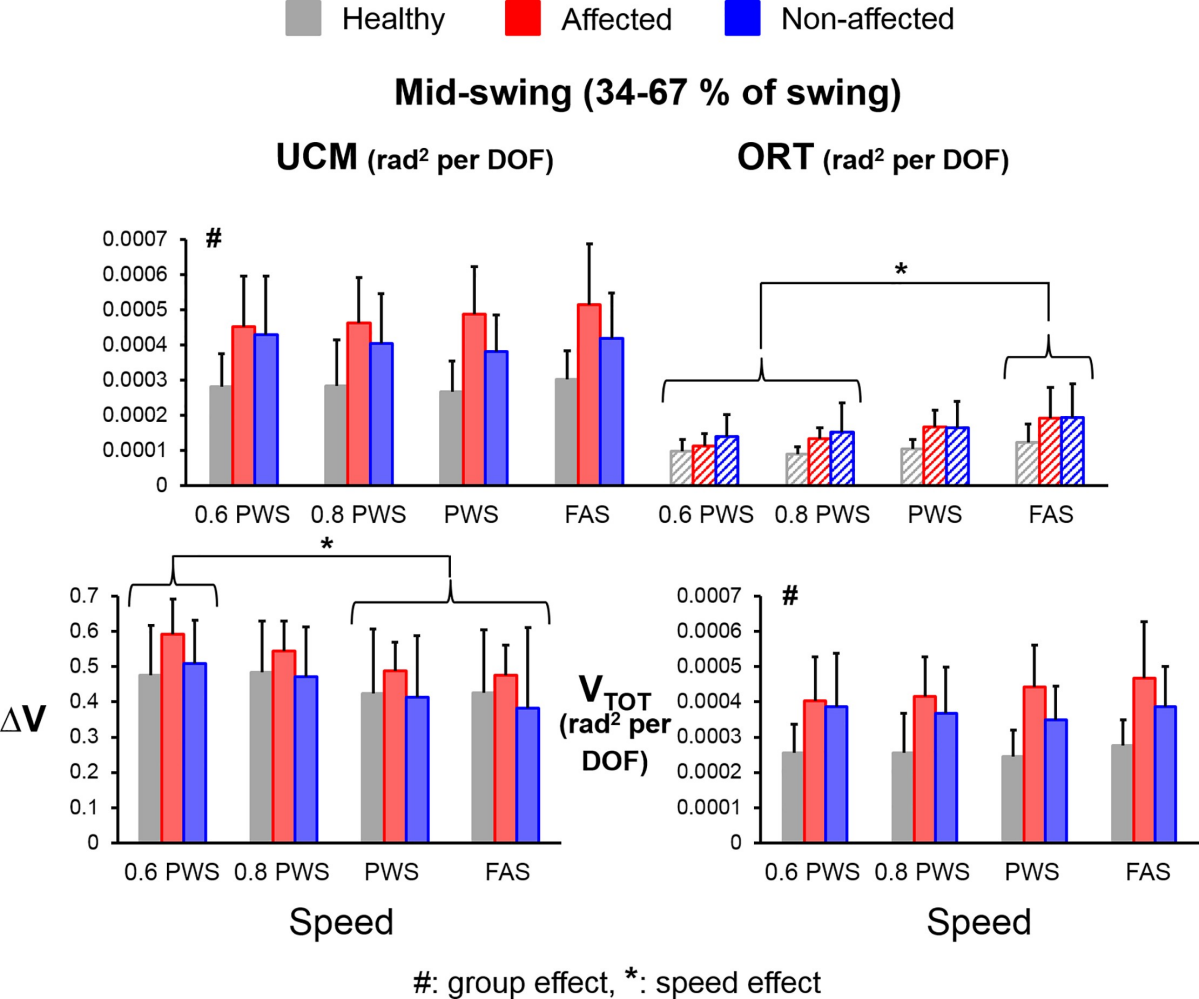
Average values of UCM variables during the mid-swing (V_UCM-early_, V_ORT-early_, ΔV_early_ and V_TOT-early_) for healthy controls (grey bars), stroke affected leg (red bars), and stroke unaffected leg (blue bars). Error bars represent 1 STD. # indicates significant difference between stroke and control groups. * indicates significant difference between different speeds.

There were significant main effects for group (F_(1,112)_ = 23.26, *η*^*2*^ = 0.17, *p*<0.001, power = 0.99) and variance component (V_UCM_ versus V_ORT_) (F_(1,112)_ = 139.61, *η*^*2*^ = 0.55, *p*<0.001, power = 1.00) as well as a significant interaction effect for group*variance component (F_(1,112)_ = 11.99, *η*^*2*^ = 0.10, *p*<0.001, power = 0.93). Both groups had significantly greater average V_UCM_ compared to average V_ORT_ during the mid-swing (V_UCM-mid_ > V_ORT-mid_) (THSD post hoc, *p*<0.05, stroke UCM-ORT: Cohen’s d = 5.09, healthy UCM-ORT: Cohen’s d = 2.78) (**Fig 5**). Compared to the healthy controls, stroke subjects had significantly greater V_UCM-mid_ (THSD post hoc, *p*<0.05, Cohen’s d = 2.76) and V_TOT-mid_ (main group effect, F_(1,48)_ = 34.59, *η*^*2*^ = 0.42, *p*<0.001, power = 1.00). There was no group effect for V_ORT-mid_ or ΔV_mid_. For the speed effect, similar to the trends at early swing, subjects also had significantly greater V_ORT-mid_ at the FAS than at the lower speeds (60% PWS and 80% PWS) (THSD post hoc, *p*<0.05, both Cohen’s d > 1.47, respectively) while having significantly greater ΔV_mid_ at the lower speed (60% PWS) than at the higher speeds (PWS and FAS) (THSD post hoc, *p*<0.05, both Cohen’s d > 1.41). There was no speed effect for V_UCM-mid_ or V_TOT-mid_.

### Late swing

**Fig 6:**
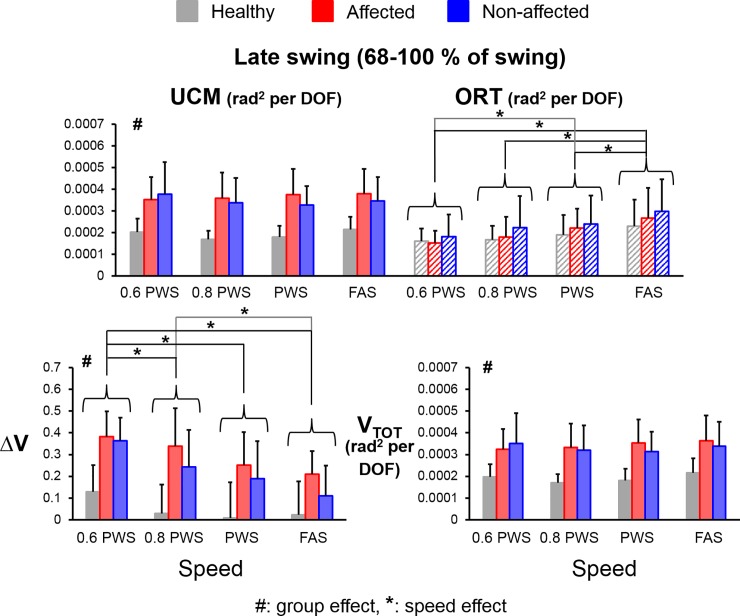
Average values of UCM variables during the late swing (V_UCM-late_, V_ORT-late_, ΔV_late_ and V_TOT-late_) for healthy controls (grey bars), stroke affected leg (red bars), and stroke unaffected leg (blue bars). Error bars represent 1 STD. # indicates significant difference between stroke and control groups. * indicates significant difference between different speeds.

There were significant main effects for group (F_(1,112)_ = 24.82, *η*^*2*^ = 0.18, *p*<0.001, power = 0.99), variance component (V_UCM_ versus V_ORT_) (F_(1,112)_ = 45.52, *η*^*2*^ = 0.29, *p*<0.001, power = 1.00), and speed (F_(3,112)_ = 7.56, *η*^*2*^ = 0.17, *p*<0.001, power = 0.98) as well as significant interaction effects for group*variance component (F_(1,112)_ = 18.81, *η*^*2*^ = 0.14, *p*<0.001, power = 0.99) and speed*variance component (F_(3,112)_ = 4.84, *η*^*2*^ = 0.11, *p*<0.01, power = 0.90). Stroke subjects had significantly greater average V_UCM_ compared to average V_ORT_ during the late swing (V_UCM-late_ > V_ORT-late_) (THSD post hoc, *p*<0.05, Cohen’s d = 3.69) (**Fig 6**). However, healthy controls had similar amount of V_UCM-late_ compared to V_ORT-late_. These results suggest that healthy subjects did not stabilize the mediolateral foot positions (Foot_ML_) during the late swing but stroke subjects still tried to stabilize their Foot_ML_ at late swing. Compared to the healthy controls, stroke subjects had significantly greater V_UCM-late_ (THSD post hoc, *p*<0.05, Cohen’s d = 3.11), ΔV_late_ (main group effect, F_(1,48)_ = 60.51, *η*^*2*^ = 0.56, *p*<0.001, power = 1.00) and V_TOT-late_ (main group effect, F_(1,48)_ = 59.55, *η*^*2*^ = 0.55, *p*<0.001, power = 1.00). There was no group effect for V_ORT-late_. For the speed effect, similar to the trends at the early and mid-swing, subjects also had significantly greater V_ORT-late_ at the higher speeds than at the lower speeds (THSD post hoc, *p*<0.05, all Cohen’s d > 0.86) whereas subjects had significantly greater ΔV_late_ at the lower speeds than at the higher speeds (THSD post hoc, *p*<0.05, all Cohen’s d > 1.10).

### Comparisons between sub-phases

In average ΔV, there were a significant main effect for group (F_(1,194)_ = 89.42, *η*^*2*^ = 0.32, *p*<0.001, power = 1.00), phase (F_(2,194)_ = 168.48, *η*^*2*^ = 0.63, *p*<0.001, power = 1.00) and a significant interaction effect for group*phase (F_(2,194)_ = 10.23, *η*^*2*^ = 0.10, *p*<0.001, power = 0.98). Healthy subjects had the greatest amount of ΔV at the mid-swing, then at the early swing and had the least amount of ΔV at the late swing (ΔV_mid_ > ΔV_early_ > ΔV_late_) (THSD post hoc, *p*<0.05, ΔV_mid_-ΔV_early_: Cohen’s d = 2.75, ΔV_mid_-ΔV_late_: Cohen’s d = 3.65, ΔV_early_-ΔV_late_: Cohen’s d = 0.89). Similar to the healthy controls, stroke subjects also had significantly greater ΔV at the mid-swing but had no difference in ΔV between the early and late swing (ΔV_mid_ > ΔV_early_ = ΔV_late_) (ΔV_mid_-ΔV_early_: Cohen’s d = 1.84, ΔV_mid_-ΔV_late_: Cohen’s d = 2.15, ΔV_early_-ΔV_late_: Cohen’s d = 0.31). These results indicate that the kinematic synergy in stabilizing the mediolateral foot positions is strongest at the mid-swing but weakest at the late swing in healthy gait. However, this phase-dependent strategy is preserved for the mid-swing but not for the late swing in the stroke subjects.

### Correlation between UCM and walking stability measures

As mentioned above, stroke subjects had significantly greater average V_UCM_, ΔV and V_TOT_ but slightly greater average V_ORT_ across the entire swing phase compared to healthy controls. In addition, stroke subjects demonstrated more local and orbital instability than the healthy controls [4]. When including both stroke and healthy data into the correlation analyses, V_UCM_, V_ORT_, ΔV and V_TOT_ are significantly, positively correlated with majority of the stability and variability measures except step width variability that is negatively correlated with ΔV (**Table 1**).

**Table 1:** Pearson’s correlation coefficients (*r*) between UCM measures and the walking stability measures.

Stability measures	UCM measures	All		Healthy		Stroke	
		*r*	p-value	*r*	p-value	*r*	p-value
Short-term LDE	V_UCM_	**0.61**	< 0.001	0.09	0.62	**0.34**	0.04
	V_ORT_	0.16	0.18	0.02	0.89	0.06	0.75
	ΔV	**0.48**	< 0.001	0.12	0.49	0.31	0.07
	V_TOT_	**0.59**	< 0.001	0.08	0.63	0.32	0.06
maxFM	V_UCM_	**0.36**	0.002	0.02	0.92	0.32	0.06
	V_ORT_	**0.36**	0.002	**0.58**	< 0.001	0.09	0.59
	ΔV	0.001	0.99	**-0.48**	0.003	0.14	0.41
	V_TOT_	**0.37**	0.001	0.09	0.60	0.30	0.07
meanSD C7 position	V_UCM_	**0.68**	< 0.001	0.14	0.40	**0.58**	< 0.001
	V_ORT_	**0.41**	< 0.001	**0.58**	< 0.001	0.32	0.06
	ΔV	**0.24**	0.04	**-0.47**	0.004	0.07	0.67
	V_TOT_	**0.68**	< 0.001	0.21	0.22	**0.57**	< 0.001
meanSD C7 velocity	V_UCM_	**0.60**	< 0.001	0.10	0.58	**0.56**	< 0.001
	V_ORT_	0.12	0.33	-0.12	0.50	0.12	0.50
	ΔV	**0.39**	< 0.001	0.01	0.94	**0.34**	0.04
	V_TOT_	**0.58**	< 0.001	0.08	0.66	**0.53**	< 0.001
mean MOS_ML_	V_UCM_	**0.33**	< 0.001	0.29	0.09	0.03	0.80
	V_ORT_	-0.06	0.56	-0.16	0.36	-0.18	0.13
	ΔV	**0.39**	< 0.001	0.20	0.24	**0.24**	0.05
	V_TOT_	**0.30**	0.001	0.25	0.14	0.01	0.96
STD MOS_ML_	V_UCM_	**0.74**	< 0.001	**0.60**	< 0.001	**0.66**	< 0.001
	V_ORT_	**0.42**	< 0.001	**0.48**	0.004	**0.40**	0.001
	ΔV	**0.20**	0.03	0.01	0.97	-0.06	0.64
	V_TOT_	**0.74**	< 0.001	**0.63**	< 0.001	**0.65**	< 0.001
mean step width	V_UCM_	**0.42**	< 0.001	-0.19	0.26	0.18	0.15
	V_ORT_	**0.23**	0.01	0.22	0.19	0.18	0.14
	ΔV	0.17	0.07	**-0.49**	0.002	-0.08	0.48
	V_TOT_	**0.42**	< 0.001	-0.16	0.37	0.18	0.12
STD step width	V_UCM_	**0.54**	< 0.001	**0.40**	0.02	**0.63**	< 0.001
	V_ORT_	**0.79**	< 0.001	**0.90**	< 0.001	**0.74**	< 0.001
	ΔV	**-0.39**	< 0.001	**-0.55**	< 0.001	**-0.48**	< 0.001
	V_TOT_	**0.59**	< 0.001	**0.50**	0.002	**0.67**	< 0.001

Note that a larger value of short-term LDE or maxFM indicates greater instability of the trunk motion represented by the C7 vertebral marker velocity profile in the mediolateral direction.

Short-term LDE: short-term local divergence exponent; maxFM: maximum Floquet multipliers; meanSD C7 position and meanSD C7 velocity: the mean variability of C7 marker positions and velocities across the gait cycle in the mediolateral direction; mean and STD MOS_ML_: mean and variability of the dynamic margins of stability in the mediolateral direction; mean and STD step width: mean and variability of step width

Stroke and healthy subjects demonstrated different relationships between UCM and walking stability measures. In the healthy control group, ΔV is significantly, negatively correlated with maxFM, C7 marker position variability, and average step width. In addition, healthy controls had V_ORT_ positively correlated with maxFM and C7 marker position variability. On the contrary, in the stroke group, either V_UCM,_ ΔV or V_TOT_ is significantly, positively correlated with short-term LDE, the variability in C7 marker position and velocity as well as the average MOS_ML_. For the variability in MOS_ML_ and step width, both stroke and healthy groups of subjects demonstrated similar relationships to UCM measures. More variances (either V_UCM_, V_ORT_, or V_TOT_) in both groups are associated with greater variability in MOS_ML_ and step width. However, greater ΔV in both groups is associated with less step width variability.

## Discussion

The current findings support our hypothesis that post-stroke individuals still possessed the capability of coordinating joint motions to stabilize the mediolateral footpath of the swing leg during walking. We found that post-stroke individuals had significantly greater amount of good variance compared to the bad variance in the mediolateral footpath stabilization, indicating that mediolateral footpath during swing is an important task variable stabilized by the CNS. Consistent to the previous findings [19, 20], our results also suggest that post-stroke individuals were able to adapt to their altered sensorimotor system to walk in a way such that the important task variable can be stabilized during walking.

In contrast to our expectation, post-stroke individuals used a stronger kinematic synergy (i.e., greater ΔV) for footpath stabilization in the mediolateral (ML) direction compared to their healthy controls. We found that post-stroke individuals had a significantly greater ΔV than the healthy controls across the swing phase. Our previous study [19] showed that there was no significant difference in the strength of the kinematic synergy to control vertical or anterior-posterior (AP) footpath between healthy and post-stroke individuals. However, it is possible that the control strategies for footpath stabilization in the AP direction may be different than those in the ML direction, with the ML footpath control requiring more active feedback [9]. Therefore, post-stroke individuals may need to significantly alter their ML footpath control strategy to compensate for the neuro-motor impairments during walking. In addition, we found that stroke subjects had significantly greater ΔV than healthy controls specifically at the late swing while healthy controls did not stabilize the ML foot positions during late swing. Consistent with the findings of Krishnan et al. (2013) on healthy gait [14], the kinematic synergy in stabilizing the ML foot positions is strongest at the mid-swing but weakest at the late swing. In addition, we also found that this kinematic synergy in footpath stabilization is stronger at the slower speeds than at the higher speeds. These results indicate that stroke subjects carefully stabilized their foot positions even at the end of swing phase, prior to the heel strikes, suggesting that they walked more cautiously than healthy controls and particularly, at the slower speed. Please note that the speed conditions tested in this study were based on the preferred walking speed (PWS) of each subject instead of using matched speeds between groups and stroke subjects had slower PWS than healthy controls [4]. This factor might have resulted in overestimating the group effect we found for the strength of the kinematic synergy. It is also possible that decreasing preferred walking speed following stroke would allow stroke subjects to precisely stabilize their foot positions during walking.

We also found that there were different relationships between UCM and walking stability measures in stroke compared to healthy subjects. In healthy subjects, a stronger kinematic synergy is associated with better orbital stability, less lateral drift on the treadmill and narrower step width. Similar relationships in healthy subjects were also seen where better stability is associated with smaller “bad variance” (V_ORT_). Thus, using a stronger kinematic synergy or minimizing “bad variance” in footpath stabilization in healthy controls would help improve walking stability. However, in stroke subjects, stronger kinematic synergy or more “good variance” is associated with less local stability, more lateral drift on the treadmill and greater variability of mediolateral trunk movement. In addition, we also found that stroke subjects demonstrated an increase in their total variance (V_TOT_) and “good variance” (V_UCM_) while maintaining similar “bad variance” (V_ORT_) in comparison to healthy controls. Previous literature has shown similar trends of increased “good variance” without changing the “bad variance” during walking following neurological injury [21], suggesting that individuals with neurological disorder employ a different control strategy than healthy controls to account for the increased movement variability. However, walking with too much “good variance” or stronger kinematic synergy in the post-stroke individuals, despite no effect on the footpath, may adversely affect overall walking stability to some extent. The current findings indicate that footpath stabilization is an important task variable that can influence walking stability in both healthy and post-stroke individuals. Given that CNS controls multiple task variables during the swing phase of walking [19, 32], future studies are warranted to further understand the alteration in the control strategies to utilize motor abundance and maintain walking stability following stroke.

Contrary to our findings, previous studies investigating multi-finger force production, arm reaching/pointing and standing balance tasks suggested that more “good variance”, less “bad variance”, or stronger kinematic synergy in task variable stabilization has the tendency of correlating with better task performance [33–35]. It is possible that we observed this disagreement with previous literature because we did not provide subjects with visual feedback on their foot positions and we did not ask them to track specific foot placement targets. Instead, our study examined steady-state walking that is more a dynamic task, requiring relatively small amount of the active feedback control for the frontal-plane stability by consuming ~20% of the metabolic energy during walking [8], whereas high-precision tasks such as arm reaching/pointing would heavily rely on active feedback control. In addition, the ability or flexibility to coordinate multiple degrees of freedom in stabilizing task variables is particularly important during unpredictable situations (e.g., encountering unexpected perturbations) compared to the predictable situations [36]. Whether the stronger kinematic synergy in footpath stabilization can help post-stroke individuals maintain walking stability during unpredictable situations will require further investigation.

## Conclusions

The current study applied UCM approach to investigate how post-stroke and neurologically intact individuals coordinate joint motions to stabilize mediolateral footpath of the swing leg and examined how the kinematic synergy in footpath stabilization correlated to their walking stability.

Stroke subjects used a stronger kinematic synergy in footpath stabilization, in particular, during late swing compared to healthy controls and at slower walking speeds. Different relationships between UCM and walking stability measures were observed in stroke versus healthy gaits. The current findings suggest that footpath stabilization is an important strategy to minimize step variability and maintain dynamic stability. However, walking with too much “good variance” in people following stroke, despite no effect on the footpath, may adversely affect their overall walking stability to some extent. To achieve a more stable walking, gait training following stroke should focus on increasing walking speeds and reducing the use of compensatory movement patterns that incorporate excessive degrees of freedom in joint motion. The current study examined steady state treadmill walking. Whether the stronger kinematic synergy in footpath stabilization could help post-stroke individuals maintain walking stability during unpredictable situations will require further investigation.
